# (*R*)-*N*-{2-*tert*-Butyl-2-[(*R*)-*tert*-butyl­sulfonamido]ethylidene}-*tert*-butane­sulfonamide

**DOI:** 10.1107/S1600536808028225

**Published:** 2008-09-13

**Authors:** Yu Hu, Xiao-Xia Sun, Cong-Bin Fan

**Affiliations:** a Experimental Chemistry Center, Nanchang University, Nanchang 330031, People’s Republic of China; b Jiangxi Key Laboratory of Organic Chemistry, Jiangxi Science and Technology Normal University, Nanchang 330013, People’s Republic of China

## Abstract

The title compound, C_14_H_30_N_2_O_2_S_2_, is the product of the monoaddition reaction of *tert*-butyl magnesium chloride with bis-[(*R*)-*N*-*tert*-butanesulfinyl]ethanediimine. There are two almost identical mol­ecules in the asymmetric unit, the mol­ecular conformation of which is stabilized by an intra­molecular N—H⋯N hydrogen bond.

## Related literature

 For general background, see: Sun *et al.* (2005 ▶). Alexakis *et al.* (2000 ▶); Alvaro *et al.* (1997 ▶). For related structures, see: Bam­bridge *et al.* (1994 ▶); Lucet *et al.* (1998 ▶); Roland & Mangeney (2000 ▶); Roland *et al.* (1999 ▶).
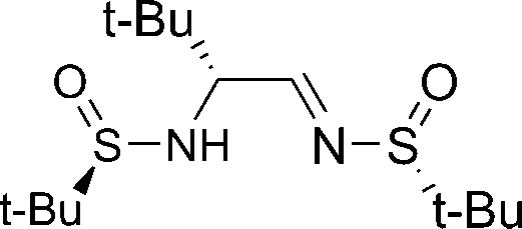


## Experimental

### 

#### Crystal data

C_14_H_30_N_2_O_2_S_2_
*M* *_r_* = 322.52
Monoclinic, 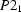

*a* = 9.714 (2) Å
*b* = 18.489 (3) Å
*c* = 11.169 (2) Å
β = 109.23 (1)°
*V* = 1894.0 (7) Å^3^
*Z* = 4
Mo *K*α radiation
μ = 0.28 mm^−1^
*T* = 291 (2) K
0.52 × 0.42 × 0.38 mm

#### Data collection

Siemens P4 diffractometer
Absorption correction: multi-scan (*SADABS*; Sheldrick, 1996 ▶) *T*_min_ = 0.936, *T*_max_ = 0.975 (expected range = 0.862–0.897)
7793 measured reflections
6860 independent reflections
5010 reflections with *I* > 2σ(*I*)
*R*_int_ = 0.020
3 standard reflections every 97 reflections intensity decay: 3.1%

#### Refinement

*R*[*F* ^2^ > 2σ(*F* ^2^)] = 0.039
*wR*(*F* ^2^) = 0.084
*S* = 0.93
6860 reflections
388 parameters
3 restraints
H atoms treated by a mixture of independent and constrained refinement
Δρ_max_ = 0.18 e Å^−3^
Δρ_min_ = −0.14 e Å^−3^
Absolute structure: Flack (1983 ▶), 3214 Friedel pairs
Flack parameter: −0.04 (5)

### 

Data collection: *XSCANS* (Siemens, 1994 ▶); cell refinement: *XSCANS*; data reduction: *SHELXTL* (Sheldrick, 2008 ▶); program(s) used to solve structure: *SHELXS97* (Sheldrick, 2008 ▶); program(s) used to refine structure: *SHELXL97* (Sheldrick, 2008 ▶); molecular graphics: *SHELXTL*; software used to prepare material for publication: *SHELXTL*.

## Supplementary Material

Crystal structure: contains datablocks I, global. DOI: 10.1107/S1600536808028225/bt2777sup1.cif

Structure factors: contains datablocks I. DOI: 10.1107/S1600536808028225/bt2777Isup2.hkl

Additional supplementary materials:  crystallographic information; 3D view; checkCIF report

## Figures and Tables

**Table 1:** Hydrogen-bond geometry (Å, °)

*D*—H⋯*A*	*D*—H	H⋯*A*	*D*⋯*A*	*D*—H⋯*A*
N1—H1*N*⋯N2	0.84 (2)	2.19 (2)	2.697 (3)	118.5 (17)
N1′—H1′*N*⋯N2′	0.84 (2)	2.18 (3)	2.672 (3)	118 (2)

**Fig. 1.**
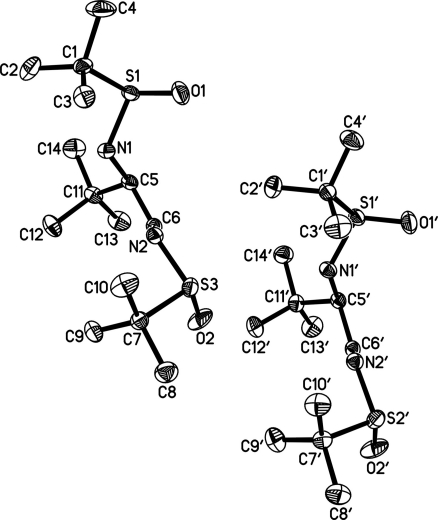
Molecular structure of title compound in the solid state with 50% probability ellipsoids showing the labelling scheme. H atoms omitted for clarity.

**Fig. 2.**
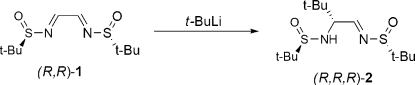
Synthesis of the title compound.

## supplementary crystallographic information

### Comment

The title compound is an important imtermediate for the synthesis of unsymmetrically disubstituted 1,2-diamines and C_2_-symmetric vicinal diamines. There are two almost identical molecules in the asymmetric unit whose molecular conformation is stabilized by an intramolecular N—H···N hydrogen bond.

### Experimental

R-1-Amino-1-tert-butyl-N,N'-bis[(R)- N-tert-butanesulfinyl]-2-iminoethane was prepared from bis-[(R)-N-tert-butanesulfinyl]ethanediimine (264 mg, 1.00 mmol). The solution of bis-[(R)-N-tert-butanesulfinyl]ethanediimine was cooled to 195 K under a argon atmosphere. 3 mol/l t-BuMgCl in diethyl ether (0.5 ml) was added slowly to the solution and stirred for 3–5 h. The combined organic layers were dried over magnesium sulfate, filtered and concentrated.

Single crystals suitable for X-ray diffraction analysis were obtained by slow diffusion of diethyl ether into the solution.

^1^HNMR (300 MHz, CDCl_3_, TMS): δ 1.03 (s, 9H, –3CH_3_), 1.22 (s, 9H, –3CH_3_), 1.26 (s, 9H, –3CH_3_), 3.98 (m, 1H, –NH), 4.84 (d, 1H, *J* = 5.6 Hz, –CH), 8.28 (d, 1H, *J* = 2.9 Hz, –CH); ^13^C NMR (75 MHz, CDCl_3_): δ  22.55, 22.78, 26.64, 36.58, 56.41, 57.12, 66.70, 168.39; FT–IR (KBr, cm-1): 1065, 1070, 1621, 3278.

### Refinement

Hydrogen atoms bonded to C were positioned geometrically and refined using a riding model with fixed individual displacement parameters [U(H) = 1.2U_eq_(C) or U(H) = 1.5 U_eq_(C_methyl_)] using a riding model with C_sp2_—H = 0.95 Å, tertiary C—H = 0.98 Å, or methyl C—H = 0.96 Å, respectively. The methyl groups were allowed to rotate but not to tip. The H atoms bonded to N were refined isotropically with a distance restraint of 0.84 (1) Å.

### Crystal data

**Table:**

C_14_H_30_N_2_O_2_S_2_	*F*(000) = 704
*M**_r_* = 322.52	*D*_x_ = 1.131 Mg m^−^^3^
Monoclinic, *P*2_1_	Mo *K*α radiation, λ = 0.71073 Å
*a* = 9.714 (2) Å	Cell parameters from 33 reflections
*b* = 18.489 (3) Å	θ = 4.4–14.3°
*c* = 11.169 (2) Å	µ = 0.29 mm^−^^1^
β = 109.23 (1)°	*T* = 291 K
*V* = 1894.0 (7) Å^3^	Block, colourless
*Z* = 4	0.52 × 0.42 × 0.38 mm

### Data collection

**Table:**

Siemens P4 diffractometer	5010 reflections with *I* > 2σ(*I*)
Radiation source: normal-focus sealed tube	*R*_int_ = 0.020
graphite	θ_max_ = 25.5°, θ_min_ = 1.9°
ω scans	*h* = −11→11
Absorption correction: multi-scan (SADABS; Sheldrick, 1996)	*k* = −21→22
*T*_min_ = 0.936, *T*_max_ = 0.975	*l* = −13→12
7793 measured reflections	3 standard reflections every 97 reflections
6860 independent reflections	intensity decay: 3.1%

### Refinement

**Table:**

Refinement on *F*^2^	Hydrogen site location: inferred from neighbouring sites
Least-squares matrix: full	H atoms treated by a mixture of independent and constrained refinement
*R*[*F*^2^ > 2σ(*F*^2^)] = 0.039	*w* = 1/[σ^2^(*F*_o_^2^) + (0.0406*P*)^2^] where *P* = (*F*_o_^2^ + 2*F*_c_^2^)/3
*wR*(*F*^2^) = 0.084	(Δ/σ)_max_ = 0.001
*S* = 0.93	Δρ_max_ = 0.18 e Å^−^^3^
6860 reflections	Δρ_min_ = −0.14 e Å^−^^3^
388 parameters	Extinction correction: SHELXL97 (Sheldrick, 2008), Fc^*^=kFc[1+0.001xFc^2^λ^3^/sin(2θ)]^-1/4^
3 restraints	Extinction coefficient: 0.0019 (5)
Primary atom site location: structure-invariant direct methods	Absolute structure: Flack (1983), 3214 Friedel pairs
Secondary atom site location: difference Fourier map	Flack parameter: −0.04 (5)

### Special details

**Table:**

Geometry. All esds (except the esd in the dihedral angle between two l.s. planes) are estimated using the full covariance matrix. The cell esds are taken into account individually in the estimation of esds in distances, angles and torsion angles; correlations between esds in cell parameters are only used when they are defined by crystal symmetry. An approximate (isotropic) treatment of cell esds is used for estimating esds involving l.s. planes.
Refinement. Refinement of F^2^ against ALL reflections. The weighted R-factor wR and goodness of fit S are based on F^2^, conventional R-factors R are based on F, with F set to zero for negative F^2^. The threshold expression of F^2^ > 2sigma(F^2^) is used only for calculating R-factors(gt) etc. and is not relevant to the choice of reflections for refinement. R-factors based on F^2^ are statistically about twice as large as those based on F, and R- factors based on ALL data will be even larger.

### Fractional atomic coordinates and isotropic or equivalent isotropic displacement parameters (Å^2^)

**Table:**

	*x*	*y*	*z*	*U*_iso_*/*U*_eq_	
S1	0.62185 (9)	0.13531 (4)	0.09753 (7)	0.0524 (2)	
S3	0.76902 (9)	0.41227 (5)	0.24900 (7)	0.0597 (2)	
O1	0.7320 (3)	0.13867 (14)	0.22466 (19)	0.0841 (7)	
O2	0.6577 (3)	0.45966 (12)	0.2718 (2)	0.0877 (8)	
N1	0.5765 (3)	0.21702 (12)	0.0354 (2)	0.0438 (6)	
N2	0.6898 (3)	0.33949 (12)	0.1602 (2)	0.0483 (6)	
C1	0.7187 (4)	0.10552 (16)	−0.0102 (3)	0.0573 (8)	
C2	0.6127 (4)	0.1053 (2)	−0.1445 (3)	0.0836 (11)	
H2A	0.6594	0.0853	−0.2005	0.100*	
H2B	0.5824	0.1540	−0.1699	0.100*	
H2C	0.5291	0.0766	−0.1482	0.100*	
C3	0.8487 (3)	0.15443 (18)	0.0034 (3)	0.0688 (9)	
H3A	0.9114	0.1322	−0.0368	0.083*	
H3B	0.9017	0.1618	0.0917	0.083*	
H3C	0.8152	0.2002	−0.0362	0.083*	
C4	0.7696 (5)	0.02897 (19)	0.0361 (5)	0.1111 (17)	
H4A	0.8332	0.0310	0.1225	0.133*	
H4B	0.8210	0.0085	−0.0159	0.133*	
H4C	0.6865	−0.0005	0.0309	0.133*	
C5	0.4821 (3)	0.25941 (14)	0.0876 (3)	0.0442 (7)	
H5	0.4620	0.2294	0.1522	0.053*	
C6	0.5596 (3)	0.32531 (15)	0.1532 (2)	0.0490 (7)	
H6	0.5110	0.3570	0.1901	0.059*	
C7	0.8329 (3)	0.45638 (15)	0.1300 (3)	0.0511 (7)	
C8	0.9063 (4)	0.52479 (18)	0.1961 (3)	0.0762 (10)	
H8A	0.9412	0.5524	0.1392	0.091*	
H8B	0.9868	0.5121	0.2699	0.091*	
H8C	0.8374	0.5531	0.2209	0.091*	
C9	0.7062 (4)	0.47343 (19)	0.0140 (3)	0.0759 (10)	
H9A	0.6643	0.4292	−0.0272	0.091*	
H9B	0.7390	0.5021	−0.0431	0.091*	
H9C	0.6342	0.4999	0.0378	0.091*	
C10	0.9417 (4)	0.4065 (2)	0.1017 (4)	0.0860 (11)	
H10A	0.9910	0.4321	0.0528	0.103*	
H10B	0.8916	0.3655	0.0544	0.103*	
H10C	1.0116	0.3904	0.1798	0.103*	
C11	0.3333 (3)	0.27616 (16)	−0.0148 (3)	0.0535 (8)	
C12	0.3570 (3)	0.3235 (2)	−0.1180 (3)	0.0729 (10)	
H12A	0.4012	0.3684	−0.0815	0.087*	
H12B	0.2650	0.3331	−0.1820	0.087*	
H12C	0.4199	0.2990	−0.1553	0.087*	
C13	0.2335 (4)	0.3140 (2)	0.0462 (3)	0.0789 (10)	
H13A	0.2351	0.2882	0.1213	0.095*	
H13B	0.1358	0.3148	−0.0126	0.095*	
H13C	0.2669	0.3626	0.0682	0.095*	
C14	0.2615 (3)	0.20401 (18)	−0.0705 (4)	0.0776 (10)	
H14A	0.1650	0.2130	−0.1280	0.093*	
H14B	0.2561	0.1731	−0.0031	0.093*	
H14C	0.3186	0.1809	−0.1152	0.093*	
S1'	0.65069 (9)	0.31033 (4)	0.68082 (7)	0.0563 (2)	
S2'	0.77418 (9)	0.60677 (4)	0.72095 (7)	0.0623 (2)	
O1'	0.7423 (3)	0.31879 (14)	0.81314 (18)	0.0963 (9)	
O2'	0.6581 (3)	0.65720 (13)	0.7277 (3)	0.0954 (9)	
N1'	0.6110 (3)	0.38924 (13)	0.6060 (2)	0.0512 (6)	
N2'	0.7029 (3)	0.52435 (12)	0.6713 (2)	0.0524 (6)	
C1'	0.7708 (3)	0.27604 (16)	0.5946 (3)	0.0561 (8)	
C2'	0.6810 (4)	0.2677 (2)	0.4578 (3)	0.0813 (11)	
H2'1	0.7416	0.2508	0.4110	0.098*	
H2'2	0.6393	0.3135	0.4246	0.098*	
H2'3	0.6044	0.2334	0.4502	0.098*	
C3'	0.8974 (4)	0.3270 (2)	0.6125 (4)	0.0946 (12)	
H3'1	0.9740	0.3023	0.5924	0.113*	
H3'2	0.9329	0.3430	0.6991	0.113*	
H3'3	0.8659	0.3680	0.5576	0.113*	
C4'	0.8225 (5)	0.20221 (19)	0.6538 (4)	0.0909 (13)	
H4'1	0.8828	0.2085	0.7406	0.109*	
H4'2	0.8777	0.1788	0.6078	0.109*	
H4'3	0.7396	0.1730	0.6503	0.109*	
C5'	0.5098 (3)	0.43696 (14)	0.6406 (3)	0.0470 (7)	
H5'	0.4925	0.4164	0.7153	0.056*	
C6'	0.5765 (3)	0.51004 (15)	0.6755 (2)	0.0501 (7)	
H6'	0.5246	0.5458	0.7008	0.060*	
C7'	0.8184 (3)	0.63050 (17)	0.5785 (3)	0.0563 (8)	
C8'	0.8951 (4)	0.70297 (18)	0.6136 (4)	0.0860 (11)	
H8'1	0.9151	0.7225	0.5414	0.103*	
H8'2	0.9850	0.6964	0.6820	0.103*	
H8'3	0.8336	0.7357	0.6395	0.103*	
C9'	0.6820 (4)	0.6364 (2)	0.4637 (3)	0.0875 (11)	
H9'1	0.6433	0.5889	0.4383	0.105*	
H9'2	0.7054	0.6589	0.3954	0.105*	
H9'3	0.6107	0.6650	0.4847	0.105*	
C10'	0.9242 (4)	0.57470 (19)	0.5606 (4)	0.0842 (11)	
H10D	1.0049	0.5700	0.6378	0.101*	
H10E	0.9591	0.5898	0.4936	0.101*	
H10F	0.8755	0.5290	0.5392	0.101*	
C11'	0.3593 (3)	0.44309 (16)	0.5317 (3)	0.0534 (8)	
C12'	0.3752 (4)	0.4788 (2)	0.4167 (3)	0.0739 (10)	
H12D	0.3947	0.5294	0.4333	0.089*	
H12E	0.2866	0.4730	0.3466	0.089*	
H12F	0.4544	0.4570	0.3965	0.089*	
C13'	0.2532 (4)	0.4833 (2)	0.5827 (4)	0.0854 (11)	
H13D	0.1572	0.4816	0.5211	0.102*	
H13E	0.2835	0.5328	0.5990	0.102*	
H13F	0.2522	0.4609	0.6599	0.102*	
C14'	0.3006 (4)	0.36648 (17)	0.4980 (3)	0.0754 (10)	
H14D	0.2041	0.3687	0.4373	0.090*	
H14E	0.2976	0.3423	0.5732	0.090*	
H14F	0.3633	0.3403	0.4625	0.090*	
H1N	0.6536 (17)	0.2404 (12)	0.046 (2)	0.043 (8)*	
H1'N	0.686 (2)	0.4126 (14)	0.607 (3)	0.059 (10)*	

### Atomic displacement parameters (Å^2^)

**Table:**

	*U*^11^	*U*^22^	*U*^33^	*U*^12^	*U*^13^	*U*^23^
S1	0.0687 (5)	0.0399 (4)	0.0566 (5)	0.0111 (4)	0.0316 (4)	0.0094 (3)
S3	0.0751 (6)	0.0619 (5)	0.0430 (4)	−0.0116 (5)	0.0207 (4)	−0.0050 (4)
O1	0.1006 (17)	0.0986 (17)	0.0521 (12)	0.0434 (15)	0.0237 (13)	0.0181 (13)
O2	0.1073 (19)	0.0810 (17)	0.1057 (19)	−0.0254 (14)	0.0769 (17)	−0.0468 (14)
N1	0.0430 (15)	0.0394 (13)	0.0545 (15)	−0.0005 (12)	0.0234 (12)	0.0015 (11)
N2	0.0558 (16)	0.0456 (14)	0.0470 (14)	0.0005 (12)	0.0218 (12)	0.0029 (10)
C1	0.074 (2)	0.0438 (16)	0.0651 (19)	0.0105 (17)	0.0382 (17)	−0.0022 (16)
C2	0.097 (3)	0.088 (3)	0.076 (2)	−0.018 (2)	0.043 (2)	−0.035 (2)
C3	0.060 (2)	0.081 (3)	0.071 (2)	0.0117 (18)	0.0299 (18)	0.0029 (18)
C4	0.161 (4)	0.051 (2)	0.157 (4)	0.037 (3)	0.101 (4)	0.014 (2)
C5	0.0469 (17)	0.0418 (16)	0.0514 (17)	0.0042 (13)	0.0263 (14)	0.0074 (13)
C6	0.062 (2)	0.0476 (18)	0.0451 (16)	0.0084 (15)	0.0278 (15)	0.0036 (13)
C7	0.0535 (18)	0.0515 (17)	0.0517 (17)	−0.0040 (15)	0.0220 (15)	−0.0024 (14)
C8	0.080 (2)	0.068 (2)	0.083 (2)	−0.015 (2)	0.031 (2)	−0.0081 (19)
C9	0.095 (3)	0.074 (2)	0.053 (2)	−0.004 (2)	0.016 (2)	0.0147 (17)
C10	0.071 (2)	0.087 (3)	0.119 (3)	−0.005 (2)	0.058 (2)	−0.024 (3)
C11	0.0447 (18)	0.0539 (18)	0.0653 (19)	0.0076 (15)	0.0225 (16)	0.0109 (15)
C12	0.061 (2)	0.088 (3)	0.064 (2)	0.0156 (19)	0.0134 (17)	0.0272 (19)
C13	0.063 (2)	0.077 (2)	0.106 (3)	0.019 (2)	0.041 (2)	0.014 (2)
C14	0.052 (2)	0.075 (2)	0.097 (3)	−0.0094 (18)	0.013 (2)	−0.005 (2)
S1'	0.0768 (6)	0.0515 (5)	0.0470 (4)	0.0158 (4)	0.0288 (4)	0.0094 (4)
S2'	0.0675 (6)	0.0626 (5)	0.0584 (5)	−0.0102 (5)	0.0230 (4)	−0.0121 (4)
O1'	0.139 (2)	0.0970 (18)	0.0443 (12)	0.0514 (18)	0.0189 (13)	0.0050 (13)
O2'	0.1054 (19)	0.0705 (17)	0.141 (2)	−0.0142 (15)	0.0817 (18)	−0.0438 (15)
N1'	0.0530 (17)	0.0495 (15)	0.0553 (15)	0.0082 (14)	0.0238 (13)	0.0069 (11)
N2'	0.0523 (16)	0.0532 (15)	0.0516 (15)	0.0041 (13)	0.0168 (13)	0.0018 (12)
C1'	0.068 (2)	0.0542 (19)	0.0505 (18)	0.0160 (17)	0.0261 (16)	0.0047 (15)
C2'	0.108 (3)	0.087 (3)	0.055 (2)	0.007 (2)	0.034 (2)	−0.0077 (19)
C3'	0.068 (2)	0.095 (3)	0.125 (3)	0.007 (2)	0.038 (2)	−0.004 (3)
C4'	0.123 (3)	0.074 (2)	0.091 (3)	0.046 (2)	0.056 (3)	0.021 (2)
C5'	0.0494 (17)	0.0515 (18)	0.0452 (16)	0.0010 (14)	0.0223 (14)	0.0036 (13)
C6'	0.060 (2)	0.0524 (18)	0.0418 (16)	0.0087 (15)	0.0217 (15)	0.0009 (14)
C7'	0.0511 (17)	0.0549 (19)	0.0652 (19)	0.0032 (16)	0.0223 (16)	−0.0015 (16)
C8'	0.081 (3)	0.072 (3)	0.107 (3)	−0.005 (2)	0.033 (2)	0.008 (2)
C9'	0.076 (2)	0.107 (3)	0.076 (2)	0.006 (2)	0.019 (2)	0.021 (2)
C10'	0.083 (3)	0.082 (3)	0.108 (3)	0.007 (2)	0.059 (2)	−0.006 (2)
C11'	0.0448 (18)	0.063 (2)	0.0516 (18)	0.0070 (16)	0.0143 (15)	0.0002 (15)
C12'	0.066 (2)	0.088 (3)	0.058 (2)	0.007 (2)	0.0077 (18)	0.0159 (19)
C13'	0.062 (2)	0.095 (3)	0.099 (3)	0.021 (2)	0.025 (2)	−0.013 (2)
C14'	0.066 (2)	0.065 (2)	0.082 (2)	−0.0054 (18)	0.0073 (19)	−0.0018 (19)

### Geometric parameters (Å, °)

**Table:**

S1—O1	1.471 (2)	S1'—O1'	1.461 (2)
S1—N1	1.661 (2)	S1'—N1'	1.662 (2)
S1—C1	1.839 (3)	S1'—C1'	1.852 (3)
S3—O2	1.478 (2)	S2'—O2'	1.484 (2)
S3—N2	1.698 (2)	S2'—N2'	1.690 (3)
S3—C7	1.833 (3)	S2'—C7'	1.833 (3)
N1—C5	1.465 (3)	N1'—C5'	1.464 (3)
N1—H1N	0.838 (10)	N1'—H1'N	0.845 (10)
N2—C6	1.268 (3)	N2'—C6'	1.272 (3)
C1—C2	1.514 (4)	C1'—C2'	1.497 (4)
C1—C3	1.520 (4)	C1'—C3'	1.509 (5)
C1—C4	1.532 (4)	C1'—C4'	1.528 (4)
C2—H2A	0.9600	C2'—H2'1	0.9600
C2—H2B	0.9600	C2'—H2'2	0.9600
C2—H2C	0.9600	C2'—H2'3	0.9600
C3—H3A	0.9600	C3'—H3'1	0.9600
C3—H3B	0.9600	C3'—H3'2	0.9600
C3—H3C	0.9600	C3'—H3'3	0.9600
C4—H4A	0.9600	C4'—H4'1	0.9600
C4—H4B	0.9600	C4'—H4'2	0.9600
C4—H4C	0.9600	C4'—H4'3	0.9600
C5—C6	1.492 (4)	C5'—C6'	1.493 (4)
C5—C11	1.551 (4)	C5'—C11'	1.568 (4)
C5—H5	0.9800	C5'—H5'	0.9800
C6—H6	0.9300	C6'—H6'	0.9300
C7—C9	1.497 (4)	C7'—C9'	1.514 (4)
C7—C10	1.513 (4)	C7'—C10'	1.515 (4)
C7—C8	1.519 (4)	C7'—C8'	1.520 (4)
C8—H8A	0.9600	C8'—H8'1	0.9600
C8—H8B	0.9600	C8'—H8'2	0.9600
C8—H8C	0.9600	C8'—H8'3	0.9600
C9—H9A	0.9600	C9'—H9'1	0.9600
C9—H9B	0.9600	C9'—H9'2	0.9600
C9—H9C	0.9600	C9'—H9'3	0.9600
C10—H10A	0.9600	C10'—H10D	0.9600
C10—H10B	0.9600	C10'—H10E	0.9600
C10—H10C	0.9600	C10'—H10F	0.9600
C11—C12	1.524 (4)	C11'—C12'	1.497 (4)
C11—C13	1.525 (4)	C11'—C13'	1.525 (4)
C11—C14	1.539 (4)	C11'—C14'	1.527 (4)
C12—H12A	0.9600	C12'—H12D	0.9600
C12—H12B	0.9600	C12'—H12E	0.9600
C12—H12C	0.9600	C12'—H12F	0.9600
C13—H13A	0.9600	C13'—H13D	0.9600
C13—H13B	0.9600	C13'—H13E	0.9600
C13—H13C	0.9600	C13'—H13F	0.9600
C14—H14A	0.9600	C14'—H14D	0.9600
C14—H14B	0.9600	C14'—H14E	0.9600
C14—H14C	0.9600	C14'—H14F	0.9600
			
O1—S1—N1	111.94 (14)	O1'—S1'—N1'	112.20 (14)
O1—S1—C1	106.14 (14)	O1'—S1'—C1'	106.52 (15)
N1—S1—C1	96.97 (13)	N1'—S1'—C1'	96.92 (13)
O2—S3—N2	110.71 (13)	O2'—S2'—N2'	110.32 (13)
O2—S3—C7	107.61 (15)	O2'—S2'—C7'	107.03 (15)
N2—S3—C7	97.23 (12)	N2'—S2'—C7'	96.83 (13)
C5—N1—S1	115.78 (18)	C5'—N1'—S1'	117.44 (19)
C5—N1—H1N	108.2 (18)	C5'—N1'—H1'N	110 (2)
S1—N1—H1N	107.9 (18)	S1'—N1'—H1'N	113 (2)
C6—N2—S3	116.8 (2)	C6'—N2'—S2'	118.1 (2)
C2—C1—C3	111.8 (3)	C2'—C1'—C3'	112.1 (3)
C2—C1—C4	111.8 (3)	C2'—C1'—C4'	110.3 (3)
C3—C1—C4	110.6 (3)	C3'—C1'—C4'	111.3 (3)
C2—C1—S1	108.7 (2)	C2'—C1'—S1'	108.1 (2)
C3—C1—S1	110.1 (2)	C3'—C1'—S1'	110.1 (2)
C4—C1—S1	103.5 (2)	C4'—C1'—S1'	104.7 (2)
C1—C2—H2A	109.5	C1'—C2'—H2'1	109.5
C1—C2—H2B	109.5	C1'—C2'—H2'2	109.5
H2A—C2—H2B	109.5	H2'1—C2'—H2'2	109.5
C1—C2—H2C	109.5	C1'—C2'—H2'3	109.5
H2A—C2—H2C	109.5	H2'1—C2'—H2'3	109.5
H2B—C2—H2C	109.5	H2'2—C2'—H2'3	109.5
C1—C3—H3A	109.5	C1'—C3'—H3'1	109.5
C1—C3—H3B	109.5	C1'—C3'—H3'2	109.5
H3A—C3—H3B	109.5	H3'1—C3'—H3'2	109.5
C1—C3—H3C	109.5	C1'—C3'—H3'3	109.5
H3A—C3—H3C	109.5	H3'1—C3'—H3'3	109.5
H3B—C3—H3C	109.5	H3'2—C3'—H3'3	109.5
C1—C4—H4A	109.5	C1'—C4'—H4'1	109.5
C1—C4—H4B	109.5	C1'—C4'—H4'2	109.5
H4A—C4—H4B	109.5	H4'1—C4'—H4'2	109.5
C1—C4—H4C	109.5	C1'—C4'—H4'3	109.5
H4A—C4—H4C	109.5	H4'1—C4'—H4'3	109.5
H4B—C4—H4C	109.5	H4'2—C4'—H4'3	109.5
N1—C5—C6	110.6 (2)	N1'—C5'—C6'	110.1 (2)
N1—C5—C11	111.5 (2)	N1'—C5'—C11'	112.0 (2)
C6—C5—C11	113.3 (2)	C6'—C5'—C11'	110.5 (2)
N1—C5—H5	107.0	N1'—C5'—H5'	108.1
C6—C5—H5	107.0	C6'—C5'—H5'	108.1
C11—C5—H5	107.0	C11'—C5'—H5'	108.1
N2—C6—C5	122.1 (2)	N2'—C6'—C5'	121.3 (2)
N2—C6—H6	119.0	N2'—C6'—H6'	119.4
C5—C6—H6	119.0	C5'—C6'—H6'	119.4
C9—C7—C10	112.3 (3)	C9'—C7'—C10'	112.3 (3)
C9—C7—C8	111.5 (3)	C9'—C7'—C8'	111.9 (3)
C10—C7—C8	111.1 (3)	C10'—C7'—C8'	109.8 (3)
C9—C7—S3	110.1 (2)	C9'—C7'—S2'	111.2 (2)
C10—C7—S3	107.9 (2)	C10'—C7'—S2'	108.3 (2)
C8—C7—S3	103.6 (2)	C8'—C7'—S2'	102.8 (2)
C7—C8—H8A	109.5	C7'—C8'—H8'1	109.5
C7—C8—H8B	109.5	C7'—C8'—H8'2	109.5
H8A—C8—H8B	109.5	H8'1—C8'—H8'2	109.5
C7—C8—H8C	109.5	C7'—C8'—H8'3	109.5
H8A—C8—H8C	109.5	H8'1—C8'—H8'3	109.5
H8B—C8—H8C	109.5	H8'2—C8'—H8'3	109.5
C7—C9—H9A	109.5	C7'—C9'—H9'1	109.5
C7—C9—H9B	109.5	C7'—C9'—H9'2	109.5
H9A—C9—H9B	109.5	H9'1—C9'—H9'2	109.5
C7—C9—H9C	109.5	C7'—C9'—H9'3	109.5
H9A—C9—H9C	109.5	H9'1—C9'—H9'3	109.5
H9B—C9—H9C	109.5	H9'2—C9'—H9'3	109.5
C7—C10—H10A	109.5	C7'—C10'—H10D	109.5
C7—C10—H10B	109.5	C7'—C10'—H10E	109.5
H10A—C10—H10B	109.5	H10D—C10'—H10E	109.5
C7—C10—H10C	109.5	C7'—C10'—H10F	109.5
H10A—C10—H10C	109.5	H10D—C10'—H10F	109.5
H10B—C10—H10C	109.5	H10E—C10'—H10F	109.5
C12—C11—C13	110.6 (3)	C12'—C11'—C13'	112.2 (3)
C12—C11—C14	110.7 (3)	C12'—C11'—C14'	109.5 (3)
C13—C11—C14	107.9 (3)	C13'—C11'—C14'	107.5 (3)
C12—C11—C5	109.6 (2)	C12'—C11'—C5'	111.4 (3)
C13—C11—C5	109.7 (2)	C13'—C11'—C5'	108.5 (2)
C14—C11—C5	108.3 (2)	C14'—C11'—C5'	107.5 (2)
C11—C12—H12A	109.5	C11'—C12'—H12D	109.5
C11—C12—H12B	109.5	C11'—C12'—H12E	109.5
H12A—C12—H12B	109.5	H12D—C12'—H12E	109.5
C11—C12—H12C	109.5	C11'—C12'—H12F	109.5
H12A—C12—H12C	109.5	H12D—C12'—H12F	109.5
H12B—C12—H12C	109.5	H12E—C12'—H12F	109.5
C11—C13—H13A	109.5	C11'—C13'—H13D	109.5
C11—C13—H13B	109.5	C11'—C13'—H13E	109.5
H13A—C13—H13B	109.5	H13D—C13'—H13E	109.5
C11—C13—H13C	109.5	C11'—C13'—H13F	109.5
H13A—C13—H13C	109.5	H13D—C13'—H13F	109.5
H13B—C13—H13C	109.5	H13E—C13'—H13F	109.5
C11—C14—H14A	109.5	C11'—C14'—H14D	109.5
C11—C14—H14B	109.5	C11'—C14'—H14E	109.5
H14A—C14—H14B	109.5	H14D—C14'—H14E	109.5
C11—C14—H14C	109.5	C11'—C14'—H14F	109.5
H14A—C14—H14C	109.5	H14D—C14'—H14F	109.5
H14B—C14—H14C	109.5	H14E—C14'—H14F	109.5
			
O1—S1—N1—C5	−74.9 (2)	O1'—S1'—N1'—C5'	−71.0 (3)
C1—S1—N1—C5	174.6 (2)	C1'—S1'—N1'—C5'	178.0 (2)
O2—S3—N2—C6	−17.2 (2)	O2'—S2'—N2'—C6'	−16.3 (3)
C7—S3—N2—C6	−129.1 (2)	C7'—S2'—N2'—C6'	−127.3 (2)
O1—S1—C1—C2	−176.6 (2)	O1'—S1'—C1'—C2'	−179.2 (2)
N1—S1—C1—C2	−61.3 (2)	N1'—S1'—C1'—C2'	−63.5 (2)
O1—S1—C1—C3	−53.7 (2)	O1'—S1'—C1'—C3'	−56.5 (3)
N1—S1—C1—C3	61.5 (2)	N1'—S1'—C1'—C3'	59.1 (3)
O1—S1—C1—C4	64.4 (3)	O1'—S1'—C1'—C4'	63.2 (3)
N1—S1—C1—C4	179.7 (3)	N1'—S1'—C1'—C4'	178.8 (2)
S1—N1—C5—C6	115.5 (2)	S1'—N1'—C5'—C6'	126.5 (2)
S1—N1—C5—C11	−117.4 (2)	S1'—N1'—C5'—C11'	−110.2 (2)
S3—N2—C6—C5	−174.66 (18)	S2'—N2'—C6'—C5'	−176.3 (2)
N1—C5—C6—N2	−0.4 (3)	N1'—C5'—C6'—N2'	0.9 (4)
C11—C5—C6—N2	−126.5 (3)	C11'—C5'—C6'—N2'	−123.3 (3)
O2—S3—C7—C9	−54.5 (3)	O2'—S2'—C7'—C9'	−49.8 (3)
N2—S3—C7—C9	59.9 (2)	N2'—S2'—C7'—C9'	64.0 (3)
O2—S3—C7—C10	−177.4 (2)	O2'—S2'—C7'—C10'	−173.7 (2)
N2—S3—C7—C10	−62.9 (2)	N2'—S2'—C7'—C10'	−59.9 (2)
O2—S3—C7—C8	64.7 (2)	O2'—S2'—C7'—C8'	70.2 (2)
N2—S3—C7—C8	179.2 (2)	N2'—S2'—C7'—C8'	−176.1 (2)
N1—C5—C11—C12	−63.8 (3)	N1'—C5'—C11'—C12'	−65.0 (3)
C6—C5—C11—C12	61.8 (3)	C6'—C5'—C11'—C12'	58.1 (3)
N1—C5—C11—C13	174.6 (2)	N1'—C5'—C11'—C13'	171.0 (3)
C6—C5—C11—C13	−59.8 (3)	C6'—C5'—C11'—C13'	−65.9 (3)
N1—C5—C11—C14	57.1 (3)	N1'—C5'—C11'—C14'	54.9 (3)
C6—C5—C11—C14	−177.3 (2)	C6'—C5'—C11'—C14'	178.0 (3)

### Hydrogen-bond geometry (Å, °)

**Table:**

*D*—H···*A*	*D*—H	H···*A*	*D*···*A*	*D*—H···*A*
N1—H1N···N2	0.84 (2)	2.19 (2)	2.697 (3)	119.(2)
N1'—H1'N···N2'	0.84 (2)	2.18 (3)	2.672 (3)	118 (2)
